# Detection of Coronary Artery Fistula between the LAD and the Great Cardiac Vein on Coronary CT Angiography

**DOI:** 10.5334/jbsr.3607

**Published:** 2024-05-30

**Authors:** Ramazan Orkun Onder, Serdar Aslan

**Affiliations:** 1 Faculty of Medicine, Giresun University; 2 Faculty of Medicine, Giresun University

**Keywords:** Coronary artery fistula, left anterior descending artery, coronary computed tomography angiography

## Abstract

Coronary artery fistulas (CAFs) are abnormal communications of coronary arteries whereby venous circuits bypass the normal capillaries within the myocardium. Coronary artery-to-cardiac vein fistula is the third most common type of CAF, accounting for 7% of cases. Electrocardiographic-gated cardiac computed tomographic (CT) angiography has emerged as a noninvasive alternative method of choice for diagnosis due to its high spatial and temporal resolution and short acquisition time. Herein, we aimed to present a left anterior descending coronary artery opened into the greater cardiac vein at the distal level, consistent with a coronary artery-to-cardiac vein fistula in a 77-year-old woman.

*Teaching point:* Coronary artery CT angiography provides a detailed evaluation of the complex anatomy of coronary artery fistula without the need for invasive methods.

## Case History

**Figure 1:**
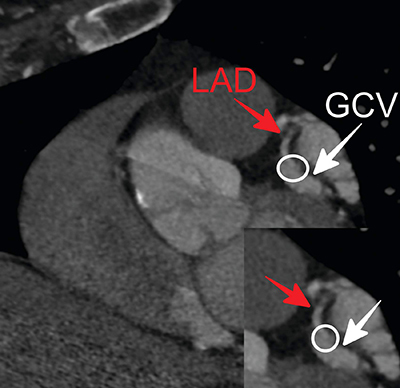
A curved planar reformatted coronary CT angiogram and magnified image show the fistulous connection (white rounds) between the left anterior descending coronary artery (red arrows) and the great cardiac vein (white arrows).

**Figure 2:**
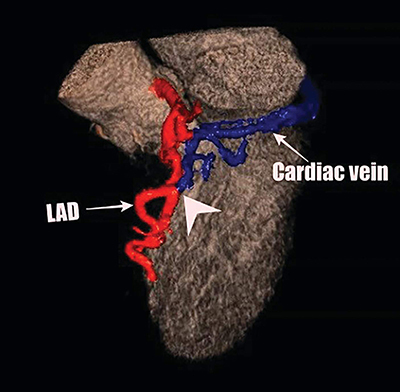
The volume-rendered 3D reconstruction and magnified image from the coronary CT angiogram show the fistulous connection (white arrowhead) between the dilated left anterior descending coronary artery (red vascular) and the great cardiac vein (blue vascular) much more demonstratively.

A 77-year-old woman presented with exertional angina for 6 months. Her medical history showed hypertension, and a physical examination detected a loud continuous heart murmur. The laboratory test results were normal. Electrocardiography showed a complete left bundle branch block. Prospective electrocardiography-gated coronary computed tomography (CT) angiography showed that the left anterior descending coronary artery opened into the greater cardiac vein at the distal level, consistent with a coronary artery-to-cardiac vein fistula (Figures 1 and 2). Invasive coronary angiography was confirmed the diagnosis, and the fistula was treated with simple ligation.

## Comments

Coronary artery-to-cardiac vein fistula is the third most common type of coronary artery fistula (CAF), accounting for 7% of cases. CAF causes a left-to-right shunt; it can overload the right side of the heart with blood, leading to pulmonary arterial hypertension and eventually right-sided congestive heart failure. While many people with small fistulas may not experience symptoms, people with larger fistulas and significant shunts may experience angina due to compromised blood flow to the heart muscle [1]. CT angiography findings of CAF include an enlarged and tortuous coronary artery terminating in dilated cardiac veins. Coronary CT angiography is an efficient and noninvasive tool for the diagnosis of this condition. In addition, multiplanar reconstruction with three-dimensional (3D) volume-rendered CT images provides a more precise assessment of the complex anatomy of the CAFs, showing the origin, course, and termination of the fistula [2]. Spontaneous closure of CAFs occurs in about 1% to 2% of cases. However, guidelines from the American College of Cardiology and the American Heart Association recommend closing symptomatic fistulas, regardless of size. Closure can be done via minimally invasive transcatheter methods or surgical ligation [3].
